# Impact of Estrogens Present in Environment on Health and Welfare of Animals

**DOI:** 10.3390/ani11072152

**Published:** 2021-07-20

**Authors:** Konrad Wojnarowski, Paweł Podobiński, Paulina Cholewińska, Jakub Smoliński, Karolina Dorobisz

**Affiliations:** 1 Institute of Animal Breeding, Wroclaw University of Environmental and Life Sciences, 51-630 Wroclaw, Poland; pawel.podobinski@upwr.edu.pl (P.P.); paulina.cholewinska@upwr.edu.pl (P.C.); 107848@student.upwr.edu.pl (J.S.); 2 Department of Otolaryngology, Head and Neck Surgery, Wrocław Medical University, 50-556 Wrocław, Poland; dorobiszkarolina@gmail.com

**Keywords:** hormones, welfare, animals, estrogens

## Abstract

**Simple Summary:**

Estrogens are a group of steroid hormones that recently have gained even more attention in the eyes of scientists. There is an ongoing discussion in the scientific community about their relevance as environmental contaminants and the danger they pose to animal health and welfare. In available literature we can find many examples of their negative effects and mechanisms that are involved with such phenomena.

**Abstract:**

Nowadays, there is a growing interest in environmental pollution; however, knowledge about this aspect is growing at an insufficient pace. There are many potential sources of environmental contamination, including sex hormones—especially estrogens. The analyzed literature shows that estrone (E1), estradiol (E2), estriol (E3), and synthetic ethinyloestradiol (EE2) are the most significant in terms of environmental impact. Potential sources of contamination are, among others, livestock farms, slaughterhouses, and large urban agglomerations. Estrogens occurring in the environment can negatively affect the organisms, such as animals, through phenomena such as feminization, dysregulation of natural processes related to reproduction, lowering the physiological condition of the organisms, disturbances in the regulation of both proapoptotic and anti-apoptotic processes, and even the occurrence of neoplastic processes thus drastically decreasing animal welfare. Unfortunately, the amount of research conducted on the negative consequences of their impact on animal organisms is many times smaller than that of humans, despite the great richness and diversity of the fauna. Therefore, there is a need for further research to help fill the gaps in our knowledge.

## 1. Introduction

Currently we can observe a growing interest in the state of the environment, methods of its protection, and the impact that the pollutants present in it have on the health of living organisms. Our knowledge of already present and emerging types of pollution is still expanding, but at an insufficient pace. Sex hormones are one of the groups of pollutants that have recently attracted the attention of scientists. The available literature indicates that the most important of them in terms of environmental impact are hormones belonging to the group of estrogens. Estrogens are a group of sex hormones that include estrone (E1), estradiol (E2), estriol (E3), estetrol (E4)—produced only during pregnancy, and often synthetic ethinyloestradiol (EE2). Estrogens are also called female hormones and they play crucial a role in female organisms, but it should be taken into consideration that they are also necessary for proper functioning of male organisms [1].

Estrogens mainly imply their effects by interaction with isoforms of the estrogen receptor (ER)—ERα and ERβ which then bind these hormones in the cytoplasm of cells and transport this complex to the cell nucleus. As a result, the activation of response elements in gene promoters begins the transcription process. Aforementioned receptors can be divided into nuclear estrogen receptors (nERs) and membrane estrogen receptors (mERs). Beside the “traditional” estrogen action, additional ways have been described—influence through cell signal transduction tied with mERs rather than genomic activity process. The available literature discusses the differences in the affinity and mechanism of action of these receptors, however, as shown by the latest studies, there is a high degree of functional and structural similarity between mERs and nERs [2]. Recently, additional receptors, namely, ER-X and Erx were described in the literature but additional research on them and mechanisms involved with their action are highly advised.

**Figure 1:**
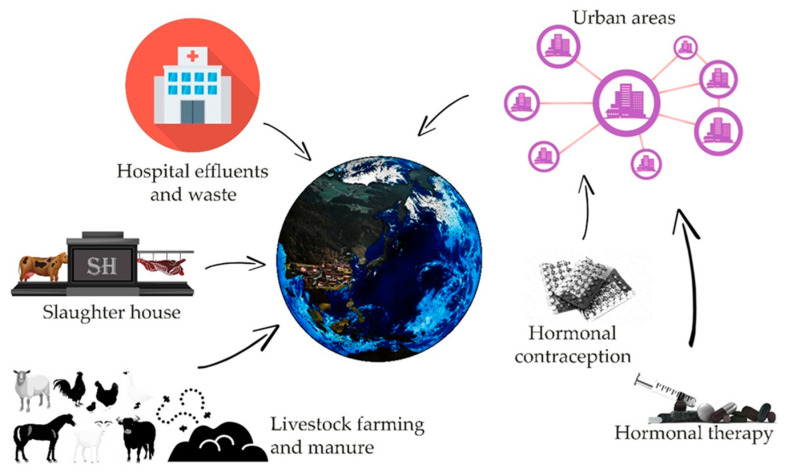
Sources of estrogens present in the environment and their simplified pathways leading to the environment (based on [4,5,6,7,8]).

There are many potential sources of contamination of the environment with estrogens, such as animal farms, slaughterhouses, or large urban agglomerations (Figure 1) [3,4]. Estrogens present in excreta, due to natural or artificial processes (like hormone therapy or contraception) contaminate, by wastewater or fertilization, water and soil. An additional factors determining the degree of risk resulting from the presence of sex hormones in the environment are their half-life, which varies depending on the physico-chemical conditions, the microbiological richness of contaminated waters and soil, from 12 h to even 180 days in the case of water reservoirs without a stabilized population of microorganisms [5]. Removal of estrogen from the aquatic environment is important, however, it is difficult to achieve even with the use of modern filtration methods [6,7,8]. As evidenced by recent research, microbial degradation of estrogens can be led by many bacteria strains i.e., *Rhodococcus, Novosphingobium*, *Acinetobacter*, *Agromyces*, and *Sphingomonas*, thus showing possible safe and inexpensive ways for the reduction of threat involved with such pollution [9,10,11,12,13]. It is worth noting that some fungi, mainly species belonging to *Aspergillus* genus are also reported to perform aerobic degradation of estrogens [9]. Another threat related to the presence of hormones in the aquatic environment may be the processes of their accumulation in bottom sediments, from which they can be released again under appropriate physical and chemical conditions [9,10,11]. Another factor of risk can be their bioaccumulation in living organisms [9,11,14,15].

Occurring in the environment, they can lead to many negative consequences for health or the functioning of organisms directly or indirectly related to it. Those effect include feminization, dysregulation of natural processes related to reproduction, deterioration of the general condition of organisms, disturbances in the regulation of apoptotic processes [16], or even promoting processes leading to cancerogenesis [17,18].

This article is the result of research on available reports and articles on the effects of estrogens in the environment and the potential threat they may pose to living organisms.

## 2. Invertebrates

Invertebrates are one of the groups most vulnerable to environmental estrogens contamination; it is related to the periodic exposure of their juvenile forms, often related to the aquatic environment, or the constant exposure of these organisms to the effects of these compounds. In the case of invertebrates, attention should be paid to a slightly different functioning of the endocrine system, both in terms of biochemistry and the mechanisms of regulation themselves [19,20], however, it does not change the fact that the presence of both natural and synthetic estrogens can affect many aspects of their lives. There are a lot of evidence in the available literature confirming the negative influence of the presence of sex hormones in invertebrates. Bovier et al. showed that the addition of EE2 solutions to the medium administered to individuals belonging to the model invertebrate species *Drosophila melanogaster* statistically significantly reduced the survival and fertility parameters of the studied insects [21].

Segner et al. [22] showed that the presence of ethinylestradiol in the living environment of *Hydra vulgaris*, *Gammarus pulex*, *Chironarus riparius*, *Hyalella Azteca,* and *Lymnaea stagnalis* may adversely affect the hatchability rate, body size, molt passing ability, reproductive behavior, and the number of eggs laid. In the case of the 1st generation of *Chironarus ripairus*, the most serious effects occurred at concentrations significantly exceeding those found in the natural environment, however, in the case of generation II, a statistically significant effect on the hatching time and an increase in the number of deformed individuals were observed. The species most sensitive to the effect of estrogens was the *Lymnea stagnalis* snail, in which statistically significant effects of ethinylestradiol on juvenile forms were observed at a concentration of 32 ng/L. The results that the juvenile stages of mollusks are most at risk of exposure to estrogens are in agreement with the results obtained by Islam et al. [23] where significantly negative effect of EE2 on the development of juvenile stages of the Australian mussel *Saccostrea glomerata* was demonstrated. The results were also consistent with those presented by Ciocan et al. [24]. In the above-mentioned study, the researchers showed that in the case of much lower concentrations of E2 (3.5–130 ng/L), they significantly influenced the expression level of ER2 and vitellogenin-associated genes in Mytilus edulis, which indicates that they presumably negatively affect the reproductive physiology of mollusks. The opposite position in the case is presented by Fodor et al. [25]; in his research, he indicated that the evidence proving the influence of sex hormones on mollusks is contradictory, and that the physiology and biochemistry of mollusks different from vertebrates cause erroneous research assumptions, and the final proof for this is the lack of specific genes related to the functioning of estrogen receptors and enzymes related to them [25].

Clubbs and Brooks [26] performed a study on *Daphnia magna* which mainly reproduces asexually. It was shown that none of the tested concentrations, i.e., 62.5, 125, 250, 500, 1000 µg/L, had a significant negative effect on the size of the F0 generation population or the growth rate of progeny. The only effect of exposure was a statistically significant increase in ovovitelin production in the group exposed to the concentration of 1000 µg/L EE2 [26], it should be noted, however, that the concentrations used in the study significantly exceeded those with which invertebrates may come into contact in the natural environment. Fernández-González et al. [27] points out that the use of ovovitelin as a potential marker of estrogen exposure is a mistaken approach and such findings should not be taken into account. Souza et al. [28] conducted studies evaluating the effect of EE2 on the activity of glutathione S transferase and Caspase-3 on the orders of Calanoida and Cyclopoida belonging to copepods. In the case of Calanoid, statistically significant differences were found in the level of glutathione S-transferase activity between the control group and the 10, 100 and 1000 ng/L groups, while with Cyclopoida statistically significant differences occurred between the control group and the 100 and 1000 ng/L groups. In the study of caspase 3 activity, statistically significant differences were found only in the case of the effect of 1000 ng/L on copepods of the Cyclopoid order. The negative effect of estrogens on invertebrates belonging to the copepod group is also confirmed by the research conducted by Marcial et al. [29] which have shown that estradiol significantly slows down the development of juvenile copepods. Ford and Leblanc [19] in their work summarizing the knowledge about the impact of endocrine disrupting compounds on invertebrate organisms have indicated that our level of knowledge has practically not changed over the last 30 years. They also concluded that some of the studies conducted so far have been based on wrong methodology. Another argument indicating the need for further research in the field is the great diversity of the world of invertebrates, and as Castro and Santos stated in their publication, the fact that estrogens have a specific effect on one invertebrate species does not mean that they will have the same effect on all other species [20]. A common conclusion resulting from the majority of publications dealing with this subject is the indication of the further need for research in this direction [19,20,30,31].

## 3. Fish

Fish, which are the most numerous and diverse group of vertebrates, due to the degree of similarity of endocrine systems functioning within this subtype to analogous systems in mammals, are significantly exposed to contamination of the environment with estrogens. In the case of fish, as in mammals, estrogens regulate behavior not only related to reproduction, but also to territorialism [32] or regulation of the immune response [33,34]. An additional risk for fish may be the process of accumulation and periodic release of estrogens to the waters from bottom sediments [14,35], as well as the phenomenon described in the literature as a mechanism “something out of nothing” resulting from the mutually reinforcing effect of estrogens and other chemical compounds present in the aquatic environment, often other chemical compounds, on the organisms of animals living in it [36].

Currently, some of such chemicals that are most widely present in water environments are those with anti-androgen properties. In studies performed by Filby et al. [37] environmental anti-androgens like flutamide have been reported to cause feminization of male fish by implying an inhibitory action on androgen negative feedback pathways which results induction of plasma vitellogenin, reduction of gonadosomatic index, and reduction of secondary sex characteristics. Golshan et al. [38] reports that vinclozolin (VZ) and bis(2-ethylhexyl) phthalate (DEHP) also cause reproductive disorders in male fish. Kinnberg et al. [39] have performed studies where adult male guppies (*Poecilia reticulata*) were exposed to flutamide, p,p′-DDE, 4-tert-octylphenol, and bisphenol which resulted in reduced number of spermatogenetic cysts and an increased number of spermatozeugmata in the ducts. This phenomenon tied with anti-androgen pollutants present in water environment may further strengthen the negative effects of environmental estrogens on fish populations. On the other hand, publication by Green et al. [40] seems to contradict that feminization of fish males involved with anti-androgens and estrogens interaction takes place in natural environment. Green states that mixtures of anti-androgens and estrogens present in British rivers have too low concentration to cause such effect, yet mixtures composed of estrogens only have caused excessive vitellogenin secretion and intersex in fathead minnow (*Pimephales promelas*) or Japanese medaka (*Oryzias latipes*).

Another important aspect may be the positive correlation between the strength of the interaction of these compounds and the environmental temperature. That is due to the link between environmental temperature and ectotherms metabolic rate [41] which in the era of global climate change may additionally increase the negative effects of the exposure of these vertebrates to estrogens. It also should be noted that Cox (negative temperature-estrogens effect correlation) and Korsgaard (positive temperature-estrogens effect correlation) have obtained opposite results in their studies [42]. In the available literature, we found many reports on the consequences of fish exposure to estrogens, such as morphological changes, behavioral, developmental, and reproductive disorders. One of the most frequently indicated effects of estrogens on fish is feminization of males of many species, manifested by changes in both primary and secondary sexual characteristics [43,44,45,46,47,48]. As a result of these changes, there may be a reduction in the number of seed cells produced or morphological changes.

Another potential negative effect of fish exposure to environmental estrogens may be a change in the gender structure of the population [43,44,49,50,51]. The presence of estrogens may also cause disturbances in the production of eggs and their quality laid by females of many species of fresh and saltwater fish, such as *Tautogolabrus adspersus*, *Pimaphales promelas*, *Oncorhynchus mykiss*, *Cyprinus carpio,* and *Danio rerio* [49,52,53,54,55]. There are also studies confirming the occurrence of disorders related to the synthesis of ovovitelin (VTG), leading to the occurrence of increased concentrations of this protein in tissues and fish serum [47,49,55,56,57,58,59]. There are publications in the literature describing estrogen-induced disorders affecting the fish population not only at the individual or species level, but also in interspecific systems. Ward et al. reports that high estrogen concentrations and temperature have disrupted predator–prey behavior between laboratory fish populations studied [60].

Studies performed by Wu et al. [61] on effects of estrone (E1) toxicity on zebrafish have shown that long-term exposure to estrone concentrations in ranges of 1–100 nM have resulted in higher rate of skeletal abnormalities in comparison to 0–0.1 nM groups. Exposure have also impacted the behavior of the fish, resulting in significant reduction of distance travelled by fish in 1 nM group in comparison to control group.

Petersen and Tollefsen [62] in their studies have also shown toxic effects of both estrone (E1) and estriol (E3) on primary culture of Rainbow trout (Oncorhynchus mykiss) hepatocytes, although effects of E1 and E3 were less potent than E2. In the given studies VTG concentration was used as a biomarker for estrogenicity. Obtained results have shown that estrogens indeed have negative effect on hepatocyte cells of fish and that in future cell cultures can be used as a future proper model for studies regarding toxic effects of estrogens on fish.

Additional example of estrogens impact on larger scale can be found in studies performed by Kidd et al. [63] where long-term exposure to low concentrations (5–6 ng/L) of estradiol (EE2) have severely impacted the fathead minnow (*Pimaphales promelas*) population. Ethinyloestradiol (EE2) have caused feminization of males, impacted gonadal development of males, and altered oogenesis in females, thus leading to near extinction of species in Experimental Lakes Area (Canada). Similar results were described in studies performed by Jobling et al., [64] where estrogens (E1, E2, and EE2) have impacted population of roach (*Rutilus rutilus*) in British rivers. Results presented by Hicks et al. [65] where upgrades to the municipal wastewater treatment plant (Grand River, Canada) and as an effect lower the amount of estrogens in environment have led to rapid decline of intersex rainbow darter (*Etheostoma caeruleum*) males strongly correspond with those presented by Kidd and Jobling. On the other hand, in studies performed by Wang et al. [66] EE2 concentrations detected in Liaodong Bay (China) 0.42 ng/L, were too low to affect Wild So-iuy Mullets (*Mugil soiuy*) population. The main reason of intersex males was found to be the presence of nonsteroidal estrogen equol in waters of the bay. There are also studies showing the occurrence of disturbances in periodic migration of fish belonging to the genus Oncorhynchus caused by the presence of estrogens in the water [67,68,69,70] which also affects fish on population level.

However, despite the whole range of publications proving the noticeable negative influence of estrogens on the system of free-living fish populations [36,47,59,71], there are a number of studies indicating the lack of observation of negative effects of this phenomenon in wild fish populations despite exposure to estrogens present in the aquatic environment [72].

The presence of such contradictory positions in the available literature indicates that the influence of sex hormones on the system of fish organisms is still not fully understood, and it is necessary to conduct further research aimed at explaining this phenomenon.

## 4. Amphibians

Among the terrestrial vertebrates, amphibians are the group most closely associated with the aquatic environment. Hence, potential exposure to estrogen contamination appears to pose a relatively greater threat to them. Most estrogens get into the environment with surface runoff or in sewage leachate, where their concentrations may be at levels that are hazardous to the health of amphibians [73,74]. In recent years, researchers have increasingly suggested that the current global decline in the amphibian population is related to the increase in pollutants, especially those of the nature of steroid hormones [63,75,76]. This phenomenon is increasingly dangerous because of the wide range of estrogenic effects on various development stages. The observed effects of pollution may lead to behavioral or sensory changes, as well as physiological changes, disrupting ontogenesis at its various stages and even being lethal [77]. In the research, the most commonly used amphibians are Anura, with the clawed frog *Xenopus laevis* adopted as the model species; also, numerous representatives of the genus Rana and Bufo.

Hoffman and Kloas [73] have focused on the analysis of the influence of EE2 on the mating behavior of *X. laevis* males. The frequency and nature of mating sounds were studied. Both at environmental and lower concentrations, a decrease in the frequency of sounds was shown; on the other hand, the frequency of “grinding”—typical for unaroused males—increased. Additional analysis of the acoustic spectrum and time parameters confirmed that as a result of EE2 exposure, the quality of the sounds produced significantly decreased. In the selection experiment, males subjected to 96-h exposure to EE2 were less often preferred by females during mate selection. It is believed that abnormalities in the processing of auditory stimuli also occur in other groups of amphibians. This happens through the reaction of estrogens to semicircular shafts (torus semicircularis), which are part of the midbrain cover in amphibians [78]. As a result, males are less willing to vocalize, while females limit their mating reactions and sometimes give up copulation. The obtained results clearly indicate that exposure to estrogen pollution reduces reproductive success in amphibians. On the other hand, there are studies suggesting that the increased presence of estrogen in the environment may stimulate the olfactory sensation of amphibians. Kikuyama et al. [79] describes the enhanced response of Jacobson organ to pheromones in the presence of estrogens. It causes increased chemotaxis of males toward the source of pheromones, e.g., females.

Numerous publications describe the influence of estrogens on the course of vitellogenesis. In amphibians, the organ responsible for this process is the liver. It is also one of the most important organs where estrogen binds. Researchers agree that the increased presence of estrogens induces the expression of the vitellogen gene (VGA), which leads to the excessive secretion of vitellogenins [80,81] thus making it a reliable biological marker of exposure to estrogens exposure. The change in the level of vitellogenin concentration was demonstrated above the EE2 value of 2.96 ng/L [73]. In literature high levels of VTG in males have been linked to feminization of future offspring, abnormalities in development of male gonads, and reduced levels of testosterone in males serum [82,83,84].

Studies conducted by Falfushynska et al. [85] performed on Marsh frog (*Pelophylax ridibundus*) with utilization of 100 ng/L estrone (E1) solution have shown that wide array of indices were significantly affected. Exposure have resulted in increased levels of vitellogenin and thyrotropin in blood plasma, elevated caspase-3 level, and lowered cholinesterase activity which could imply proapoptotic activity and the level of neurotoxicity. Additionally, higher levels of the DNA strand breaks were observed than in control group.

The most frequently observed phenomenon in the case of increased estrogen levels in the environment is feminization of amphibians at various stages of development. A Canadian study conducted on the juvenile stages of *Rana septentrionalis* and *Rana clamitans* presents results related to the growth and development of amphibians treated with synthetic EE2. Park and Kidd [75] found that in the case of R. *clamitans*, hatch success was significantly reduced. While the field study showed no significant changes in growth and development, results when compared to previous years’ data showed a significant percentage of hermaphroditic tadpoles. Histological studies confirmed, however, that the concentration of EE2 in the leachate led to far-reaching disturbances in the development of gonads. The presence of oocytes on the testes (intersex gonads), atrophy of reproductive cells, or complete deformation were observed among the studied individuals. Some reports confirm the feminization and demasculinization of gonads, also pointing to the phenomenon of polygonadism (more than two gonads—sometimes up to six) (Hayes et al., 2006) [76]. The mechanism of gender differentiation in amphibians is a very complex issue. We now know that it is multifactorial in nature and occurs in two stages: genotype differentiation and gonad differentiation. In the case of *X. laevis,* during hatching, young tadpoles are exposed to androgens and E2 from the female. With further development, young frogs begin to produce the necessary hormones and their corresponding receptors on their own, the levels of which peak during metamorphosis [86]. It follows that the presence of exogenous estrogens may disrupt the process of sex differentiation at its various stages. Current research also specifies genes responsible for gonad differentiation: pglyrp2, apoa1, fgb, tdo2, ca6, nags, cpb2, tmprss6, nudc, zwilch [87]. Their action and expression of appropriate proteins may be influenced by both natural estrogens as well as other pollutants present in the environment.

## 5. Reptiles

A significant part of the publications related to estrogenic effects in reptiles concerns communal pollutants and endocrine-active compounds. Of all vertebrate groups, reptiles are least frequently tested for toxicological environmental hazards [88]. However, there are a few reports detailing the effects of estrogens at different levels of the organization.

One of the few fully sequenced estrogen-binding domains in reptiles is the green anole estrogen receptor (Anolis carolinensis). The studies conducted by Matthews et al. [89] were aimed at comparing the affinity of receptors of various vertebrate species to selected estrogenic compounds. A total of 34 substances, both natural and synthetic, were tested. The comparison showed that the estrogen receptors had a much stronger affinity for dihydrotestosterone and numerous phytoestrogens than the mammalian, avian, or fish receptors. For natural estrogens, the degree of affinity is similar, regardless of the species.

One of the biomarkers indicating the pollution of the environment with estrogens is the level of vitellogenin in the peripheral blood. Tada et al. [90] confirmed the influence of increased E2 content on the increased expression of vitellogenins in a study on Chinese turtles *Mauremys reevesii*. Similarly, the results obtained on the *Trachemys scripta* by injection of E2 and diethylstilbestrol confirmed the increased level of vitellogenins [91]. This makes it possible to control both reptiles and other vertebrates in terms of estrogen exposure. It has long been known that the synthesis of vitellogenins is mainly regulated by the level of estrogens [92]. Studies on the *Chrysemys picta* and *Sternotherus odoratus* also confirmed the direct relationship of estrogen levels and ovarian development [93].

The effect of exogenous estrogens on reptiles can be considered in various ways. This is due to the peculiarities of their biology. Not only can they live in various environmental conditions, but also their life span is relatively long, and the determination of sex is twofold—it can be genotypic as well as dependent on environmental factors [88,92]. Due to the aforementioned factors, exposure to potential pollutants may not only lead to many years of bioaccumulation, but also to morphological degeneration at every stage of development.

A study of American alligators (*Alligator mississippiensis*) from Lake Apopka, Florida, provided evidence of the estrogenic effects of agricultural pollutants on reptile development. The contaminated young female alligators were shown to have an almost doubled plasma E2 level. As a consequence, females presented abnormal ovarian structures, with supernumerary follicles and polynuclear oocytes. Young males, on the other hand, showed an underestimated testosterone level, manifested by an abnormally reduced size of the testicles and penis [94]. Later studies also showed overproduction of steroid hormones and liver degradation due to environmental pollution [95]. Alligators from Lake Apopka have been carefully studied by other researchers. Lind et al. [96] report the effect of estrogenic compounds on the disturbance of the bone structure. There was a significant increase in the density and a strong mineralization of the spongy structure of long bones, which translated into an increased skeleton weight.

## 6. Birds

In birds, as in mammals, estrogens are biosynthesized, inter alia, in the sex glands and in tissues other than the sex glands, such as skin, heart, muscles, liver, brain, adipose tissue, brain, pancreas, and adrenal glands. Estrogen plays a key role in the control of reproductive behavior and the regulation of the neuroendocrine system, and is also essential in regulating the growth and differentiation of axons and dendrites in the brain [97,98]. Chemical pollutants, including reproductive hormones, may adversely affect bird reproduction and viability, as well as the development and functions of the hypothalamic-pituitary axis [99,100].

**Figure 2:**
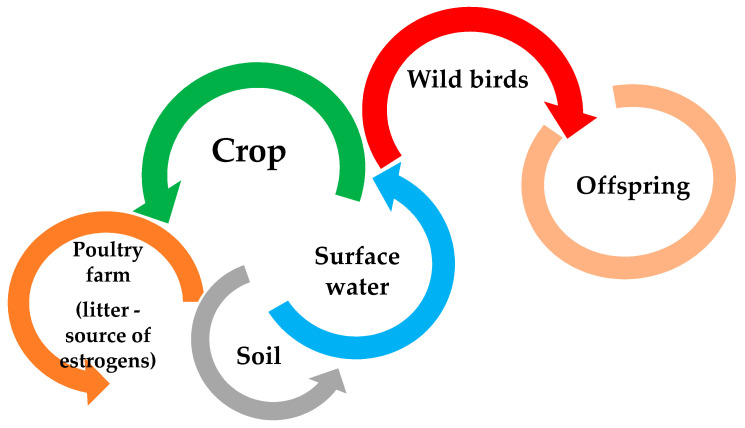
Estrogen contamination route (based on [5,102,103,105,106]).

Increasing the level of estrogens in birds reduces fertility, slows down sexual maturity of females, impairs male mating behaviors, and also accumulates in the tissues of an adult individual, as well as in eggs, which can negatively affect the development of embryos (including increasing their mortality) [100,101]. In addition, in the case of farmed birds, increasing the level of estrogen in the body may have adverse effects on the animal, but also resulted in the hypothesis that the use of their feces in farmland has recently been recognized as the main source of estrogen in the environment [4]. Poultry feces, causing contamination of surface waters with estrogens [5,102,103], negatively affect the reproductive behavior of wild birds (i.e., the perception of male sounds by females), including the development of their offspring, due to accumulation in the body (Figure 2) [104,105,106,107]. The sensitivity of birds to estrogen levels in the environment is, however, strongly correlated with the species and their age, except during development. Too high level of estrogens in the environment can also disturb the behavior of birds, often increasing the risk of aggressive behavior and impair the functioning of the immune system. On the other hand, the increased level of estrogens is also accumulated in the egg and the forming embryo, which may cause, among others: impairment of male reproductive behavior or disruption of sexual differentiation of the nervous systems that controls reproduction, and in worst case death of the embryo [4,104,106,107,108,109,110].

## 7. Mammals

Estrogens can enter mammals in many ways, not only by ingesting water contaminated with them or through the skin during contact with it [111], but also through the food they eat, an example of which may be the accumulation of estrogens along with trophic levels in consumed foods, e.g., in plants [112,113], fish [114] or even as indicated in the literature in milk, however, in this case the literature reports are contradictory [115]. A very important phenomenon in the context of the threat posed by estrogens is the fact that very often mixtures of these are found in the environment, which in addition to the additive effect of these compounds may also show the synergistic effect mentioned previously [36,116]. There are a number of potential negative consequences of their action on mammals such as reproductive disorders and lowering the general condition of the body associated with their negative impact at their excessively normal concentrations [117]. There are many reports in the literature indicating an increased risk of carcinogenesis under the influence of estrogens [5,17,115,118]. Słowikowski et al. [119] describe two mechanisms that can lead to this, i.e., by destroying the structure of proteins or the structure of the genetic code. The effect of estrogens has been associated with cancers of the prostate, lung, endometrium, and breast [5,17,115,118].

Scientists focus on the mechanism of appetite regulation and energy balance, which are influenced by estrogens. Animal studies have shown that the disturbance of estrogen levels may disturb this balance and lead to the occurrence of overweight or obesity [120,121], which in developing countries pose increasing threats to both animal and human health.

As shown in the study by Della Torre et al. [122] in mice, after administration of a dose of 17β-estradiol, they observed increased production of the alpha estrogen receptor (ERα) in bone, brain, and liver tissues. They also showed that this phenomenon is related to the differences observed during the pathophysiological processes of the liver. There is still a discussion in the scientific community about the influence of endocrine-active compounds and estrogens present in the environment on the decline in the number of normal sperm in male mammals [123,124], including humans [125], which has been observed for decades. As demonstrated by Stewart et al. [126,127], after entering the body, these compounds can induce many non-genomic and genomic cell pathways in somatic male gonads, leading to structural disorders. However, the most serious effect of the influence of estrogens is the occurrence of changes in MAPK signaling and the subcellular localization of SOX9, suppressing genes related to the development of the testicles, leading to impairment of their structure, function, and fertility. The presence of the abovementioned mechanism related to the influence of higher than physiological concentrations of estrogens on the reduction of fertility in male mammals is also indicated by Dostalova et al. [128]. Sze Yee Wee et al. [129] in their review have also pointed out that E1, E2, and E3 all have similar negative effect on fertility of males, although different in their magnitude, with E1 and E3 being least potent than E2 in their effects.

As indicated in the literature, exposure of female horses to the concentration of phytoestrogens, but, as Shemesh et al. [130] assumed in his publication, also to estrogens, may reduce the ability to bear offspring in horses, which is manifested by resorption of the fetuses [130] and the development of infertility in female sheep (Sheep Clover Disease) [130,131], however, importantly, he did not observe a similar phenomenon in the studies performed on sheep in Israel. Setchell described similar observations on estrogens present in food and related infertility in cheetahs in his publication [132]. All data regarding potential risks and roles of estrogens were summarized in Table 1.

## 8. Conclusions

The available literature clearly indicates the problem posed by estrogens in the environment. Contact with them can expose animals to many sicknesses and welfare disruptions. There is also a need for more research to help fill the gaps in our knowledge. Unfortunately, the amount of research conducted on the negative consequences of their impact on animal organisms is many times smaller than that of humans, despite the great richness and diversity of the fauna. Undoubtedly, the concentrations of estrogens present in the environment are alarming and can have far-reaching consequences for the health of animals. Particular attention should be paid here to the mechanisms of estrogen accumulation both in the environment and in animal organisms, along with the levels of the trophic chain, and potential interactions with other compounds present in the environment.

Equally alarming as the lack of complete knowledge on the negative effects of estrogens on animal health is the significant difficulty in removing estrogens present in the environment and the fact that the relevant technologies are still largely under development.

All these factors only confirm the strong need of defining estrogens as emerging contaminants and the need to focus science on the threat they currently pose.

## Figures and Tables

**Table 1:** Summary of the effects of estrogens.

Estrogen	Skeletal Formula	Role	Effects of Toxicity	References
Estrone (E1)	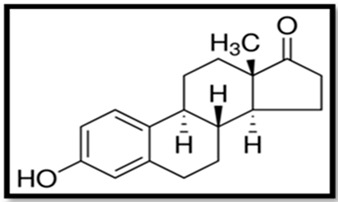	Controls the estrous or menstrual cycle in females. Has a vital role in a endocrine system of postmenopausal woman. Plays role in osteoblastogenesis.	Gonadal abnormalities, reduced fertility, males mating behavior impaired, elevated levels of thyrotropin and vitellogenin in blood plasma, elevated caspase-3 level and lowered cholinesterase activity, can induce proapoptotic and anti-apoptotic activity, neurotoxicity.	[5,62,85,129,133,134]
Estradiol (E2)	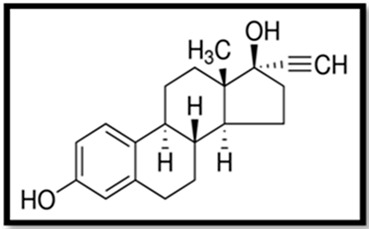	Controls the estrous or menstrual cycle in females. Regulates proper development of female reproductive system. Plays role in regulation of neuroendocrine, skeletal and immune systems in males and females. Most potent estrogen.	Gonadal abnormalities, reduced fertility, elevated level of vitellogenin in serum and tissues, can induce proapoptotic and anti-apoptotic activity, neurotoxicity. Impairs animals mating behavior, potentially cancerogenic	[25,77,94,133,135]
Estriol (E3)	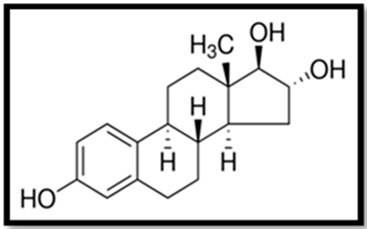	Physiological role is not fully known Regulates uteroplacental blood flow and placental vascularization	Gonadal abnormalities, reduced fertility, elevated level of vitellogenin in serum and tissues	[61,62,85,129,136,137]
Estetrol (E4)	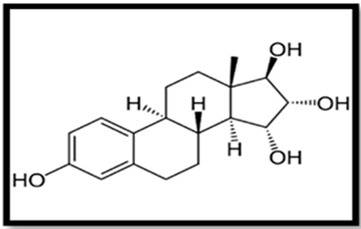	Physiological role unknown Inhibits ovulation Further research needed	Effects of toxicity are unknown May cause similar effects to other estrogens	[138,139,140]
Ethynyloestradiol (EE2)	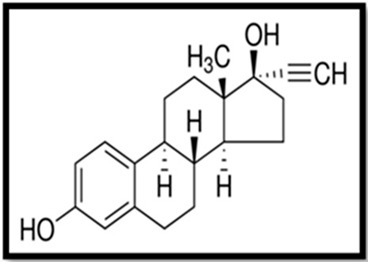	Synthetic estrogen, mainly used in contraceptive pills and hormonal therapy Inhibits ovulation	Reduces fertility, gonadal abnormalities, decreased body weight, accelerated vaginal opening, alteration of estrous cycle, impairment of behavior, formation of lesions, elevated level of vitellogenin in serum and tissues, can induce proapoptotic and anti-apoptotic activity	[5,17,70,141,142,143]

## Data Availability

Not applicable.
